# EMR-HRNet: A Multi-Scale Feature Fusion Network for Landslide Segmentation from Remote Sensing Images

**DOI:** 10.3390/s24113677

**Published:** 2024-06-06

**Authors:** Yuanhang Jin, Xiaosheng Liu, Xiaobin Huang

**Affiliations:** School of Civil Engineering and Surveying & Mapping Engineering, Jiangxi University of Science and Technology, Ganzhou 341000, China; yhjin@mail.jxust.edu.cn (Y.J.); gisbin@mail.jxust.edu.cn (X.H.)

**Keywords:** remote sensing, landslide segmentation, HRNet, attention mechanism

## Abstract

Landslides constitute a significant hazard to human life, safety and natural resources. Traditional landslide investigation methods demand considerable human effort and expertise. To address this issue, this study introduces an innovative landslide segmentation framework, EMR-HRNet, aimed at enhancing accuracy. Initially, a novel data augmentation technique, CenterRep, is proposed, not only augmenting the training dataset but also enabling the model to more effectively capture the intricate features of landslides. Furthermore, this paper integrates a RefConv and Multi-Dconv Head Transposed Attention (RMA) feature pyramid structure into the HRNet model, augmenting the model’s capacity for semantic recognition and expression at various levels. Last, the incorporation of the Dilated Efficient Multi-Scale Attention (DEMA) block substantially widens the model’s receptive field, bolstering its capability to discern local features. Rigorous evaluations on the Bijie dataset and the Sichuan and surrounding area dataset demonstrate that EMR-HRNet outperforms other advanced semantic segmentation models, achieving mIoU scores of 81.70% and 71.68%, respectively. Additionally, ablation studies conducted across the comprehensive dataset further corroborate the enhancements’ efficacy. The results indicate that EMR-HRNet excels in processing satellite and UAV remote sensing imagery, showcasing its significant potential in multi-source optical remote sensing for landslide segmentation.

## 1. Introduction

Landslides, as a typical geological disaster, pose significant risks to human life, safety and natural resources globally [1,2]. Rapid changes in the global climate and environment, particularly frequent extreme weather events, create natural conditions conducive to landslides [3]. Consequently, rapid and accurate detection of landslide areas is crucial for emergency responses and post-disaster recovery. Precise segmentation of landslide spatial information, including location and extent, forms the basis for landslide susceptibility modeling, risk assessment and other tasks [4,5].

Traditional landslide detection heavily relies on manual field surveys. Although accurate, these methods are inefficient and pose safety risks [6]. Recent advancements in remote sensing technology provide new perspectives and means for landslide detection, such as high-resolution imagery [7,8,9], Synthetic Aperture Radar (SAR) interferometry [10,11] and LiDAR [12,13,14]. These data have led scholars to propose various automatic landslide detection methods, with optical remote sensing imagery offering rich spectral information and high-resolution surface images, aiding in distinguishing landslides from other land cover types [15]. Landslide detection methods based on optical remote sensing include visual interpretation, feature thresholding, machine learning and deep learning. Visual interpretation, an early method in remote sensing, relies on expert experience and, despite its accuracy, is inefficient [16]. Feature thresholding methods, whether pixel-based or object-based, involve calculating spectral, textural, geomorphological or topographical features of landslide areas and selecting one or more thresholds for detection. For example, Ma [17] used the Normalized Differential Soil Brightness Index (NDSI), Normalized Difference Vegetation Index (NDVI) and Shadow Index (SI) to extract suspected landslide areas, excluding non-landslide areas based on slope, shape and size. Although these methods yield considerable results, they highly depend on subjectively set thresholds, making the process time-consuming and limited in automation [18].

Machine learning (ML) has shown significant potential in handling high-dimensional data and mapping complex feature categories, particularly in classification tasks. Mainstream methods include Support Vector Machines (SVMs), Decision Trees (DTs), Random Forest (RF) and Artificial Neural Networks (ANNs) [19]. Cheng [20] used a scene classification approach based on a visual bag of words, combined with an unsupervised Probabilistic Latent Semantic Analysis (PLSA) model and K-Nearest Neighbors (K-NN) classifier, to build a landslide detection model for remote sensing imagery. Although ML techniques are widely used in remote sensing, they may face overfitting or noise introduction issues when processing high-dimensional data, leading to performance degradation.

With the advancement of artificial intelligence and computational power, deep learning (DL) has achieved significant results in remote sensing image processing, especially in classification [21,22], segmentation [23,24,25] and detection [26,27,28]. Deep learning has shown immense potential in landslide detection in remote sensing imagery. Against this backdrop, Li [29] proposed a PSPNet network model based on MobileNetV2, which reduces computational complexity and enhances processing efficiency. Bui [30] initially applied convolutional neural networks to classify images containing landslides and then introduced a new image transformation algorithm to accurately detect landslide areas and sizes under different lighting conditions. Chen [31] incorporated the SENet attention mechanism into the UNet model to enhance landslide detection performance. Jin [32] not only introduced attention mechanisms into the feature extraction network for performance optimization but also included a Digital Elevation Model (DEM) and river distribution data in the dataset to strengthen model detection effectiveness. Chandra [33] explored the performance differences of using ResNet50, ResNet101, VGG-19 and DenseNet-121 as backbone models in landslide detection based on UNet. Niu [34] introduced attention modules to UNet and pruned the model to reduce the parameter count, and their proposed Reg-SA-UNet showed good results in landslide detection. Wang [35] proposed a multi-level feature enhancement network (MFENet) including the Post-Feature Enhancement Module (PFEM), Bi-Feature Difference Enhancement Module (BFDEM) and Flow Direction Calibration Module (FDCM) for successful landslide detection. Ji [36] designed a 3D CNN model with spectral and spatial attention that improves landslide detection accuracy in high-resolution remote sensing imagery by focusing on important parts in channel and spatial dimensions through attention mechanisms. Zhang [37] introduced a landslide detection network model by combining change detection and Multiple Instance Learning (MIL), using the MIL framework to reduce the need for pixel-level samples. Xia [38] enhanced landslide detection models in retaining high-resolution remote sensing image details by introducing multi-pore pyramid pooling. Ghorbanzadeh [39] used Dempster–Shafer theory combined with CNN models trained on multiple datasets to improve landslide detection accuracy.

Despite these achievements, current landslide segmentation tasks still face several challenges. Primarily, landslide remote sensing image datasets are often limited in scale and possess homogeneous labeling formats, potentially leading to data insufficiency and overfitting in models, thereby diminishing their performance in practical applications. Moreover, in comparison to natural or medical images commonly used in computer vision, remote sensing images typically present more complex scenes and richer details, rendering the extraction of effective information more challenging. Current segmentation network models, constrained by their structural limitations, exhibit restricted feature extraction capabilities, often resulting in blurred post-segmentation boundaries [40]. Additionally, the diverse textures and shapes present in landslide areas within remote sensing images further complicate identification and segmentation tasks. However, existing landslide segmentation models often fail to capture sufficient contextual information in regions with complex textures and forms, resulting in suboptimal recognition outcomes. In light of these issues, this paper introduces a novel data augmentation method and an EMR-HRNet model based on varied attention mechanisms, aimed at efficiently and automatically extracting landslide information from remote sensing images. The contributions of this paper are as follows:

1. We propose a novel data augmentation method, CenterRep, which changes the contour of landslides, enabling the model to learn more complex landslide features and improve its robustness in real-world landslide segmentation.
2. Based on HRNet [41], we introduce a network based on a feature pyramid structure. This network enhances the model’s multi-scale processing capabilities and significantly improves its ability to recognize landslide edges.
3. We incorporate a convolution block with the EMA attention mechanism, effectively enhancing the expression of spatial semantic features and achieving more accurate landslide contour recognition.

**Figure 1:**
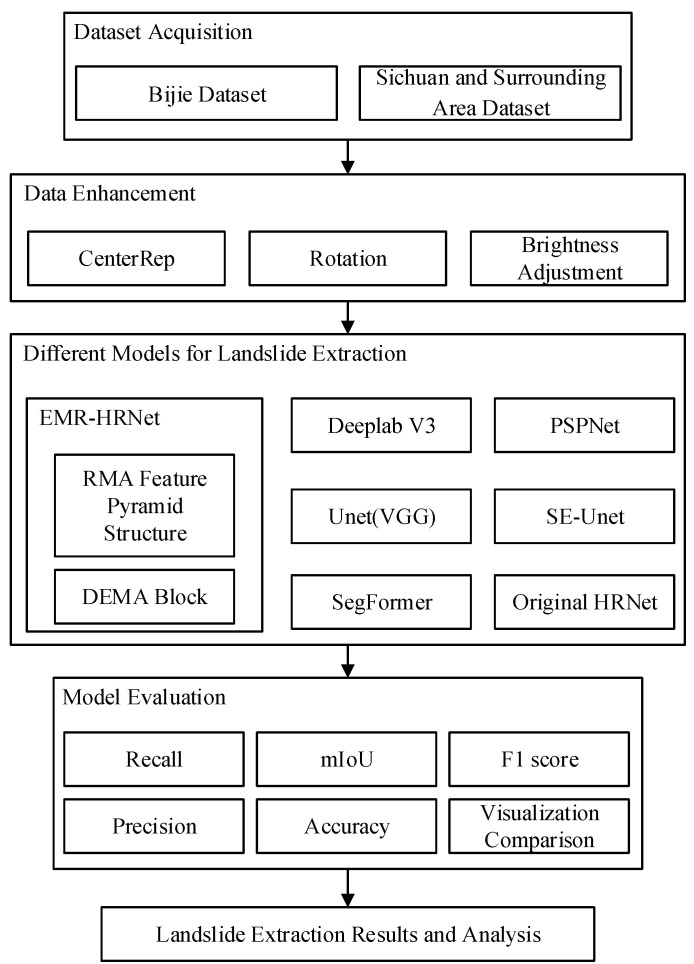
Flowchart of landslide segmentation techniques.

The rest of this article is organized as follows. Section 2 provides detailed descriptions of the datasets used in this study, as well as the data augmentation method and EMR-HRNet model proposed herein. Section 3 presents the experimental results, comparing and analyzing the proposed methods against several advanced approaches. Section 4 comprises the discussion, covering the performance of the proposed model on other datasets and efficiency comparisons with different models. Finally, Section 5 concludes the paper. The technical flowchart of this paper is shown in Figure 1.

## 2. Materials and Methods

### 2.1. Dataset

**Figure 2:**
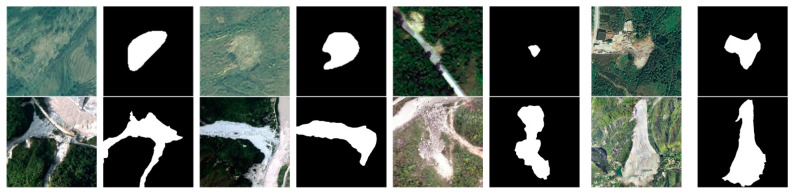
Partial landslide data image. Row 1 is the Bijie dataset, and row 2 is the Sichuan and surrounding area dataset.

To thoroughly validate the generalization ability and universality of our proposed model, two distinct datasets were employed for testing and evaluation. The primary dataset’s study area is located in Bijie City, situated in the northwestern part of Guizhou Province, China. The Bijie dataset’s imagery, acquired by the TripleSat satellite from May to August 2018, has a spatial resolution of 0.8 m. Most of the landslides in this dataset were triggered by rainfall, earthquakes and human activities [42]. The landslide types include debris avalanches, rock slides and rockfalls. As depicted in Figure 2, Bijie City is positioned on the transitional slope from the Qinghai–Tibet Plateau to the eastern hills, with a latitude of 26°21′–27°46′ N and a longitude of 103°36′–106°43′ E, covering about 26,853 square kilometers of the entire territory of Bijie City, with the terrain dominated by plateaus and mountains and with an average elevation of 1600 m. Its fragile geological environment, instability, undulating terrain and abundant rainfall (with an annual average of 849–1399 mm) categorize it as a landslide-prone area.

In addition, we incorporated a subset of high-precision aerial landslide imagery datasets, primarily from regions such as Wenchuan, Jiuzhaigou and the Jinsha River in Sichuan Province, China. The geographic coordinates of this dataset range from 97°20′50″ E to 108°32′33″ E and from 26°02′53″ N to 34°18’54″ N. This dataset features imagery with spatial resolutions ranging from 0.2 to 0.9 m and includes types of landslides such as earthquake-induced slides, rainfall-induced slides, gully debris flows and slope debris flows. The landslide locations in these areas are diverse, covering different geological and topographical backgrounds. The inclusion of these data ensures that the experimental dataset not only spans various geographical backgrounds and landslide types but also encompasses landslide imagery of multiple resolutions, thus guaranteeing the dataset’s breadth and representativeness. Through this array of diverse data, a comprehensive assessment of the proposed model’s recognition effectiveness and applicability under different environmental conditions is enabled. Table 1 presents the parameters associated with the Bijie dataset and the Sichuan and surrounding area dataset.

### 2.2. CenterRep Data Enhance

As mentioned in Section 2.1, a total of 877 different types of landslide images were obtained. This volume of data may not be sufficient to meet the training requirements of deep learning models, posing a risk of overfitting. Therefore, data augmentation is necessary. Currently, most deep learning-based landslide segmentation studies focus on optimizing model structures to enhance recognition accuracy, with insufficient attention given to the importance of data augmentation in improving model learning and recognition performance. Consequently, this paper proposes a specialized data augmentation method for landslide imagery, named CenterRep, aimed at enhancing model recognition performance by increasing the complexity of information in the data.

**Figure 3:**
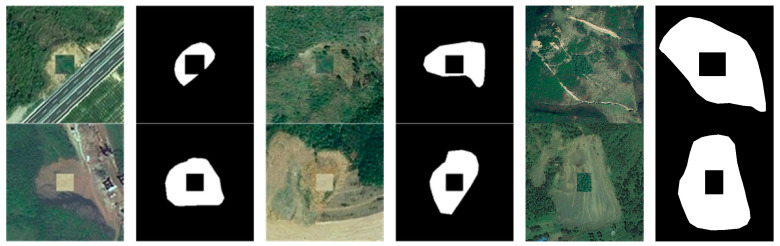
Raw images and labels enhanced with CenterRep data.

In existing landslide image datasets, including those used in this paper and by other scholars, landslide features are often marked as a completely enclosed curvilinear shape. This method of data annotation may lead to insufficient generalization capabilities during the model training process, particularly in effectively extracting landslide edge features. Therefore, with landslide labels predominantly located in the central regions of the images, this paper designs a data augmentation method named CenterRep, specifically for landslide imagery, with the hope of improving model recognition performance through specific image editing techniques. The steps are as follows: (1) Image Reading and Preprocessing. The input grayscale image is converted to the RGB mode, and the image’s width and height are obtained to facilitate subsequent pixel-by-pixel traversal to identify landslide areas. (2) Landslide Area Detection. Each pixel in the image is traversed, the landslide areas are identified, and their coordinates are recorded. (3) Calculation of Landslide Area Attributes. The bounding box of the landslide area is calculated, its maximum and minimum x and y coordinates are determined, and the center coordinates of the landslide area through the bounding box are calculated. (4) Non-landslide Feature Calculation and Overlay. Based on the width and height of the landslide area, the size of the non-landslide features to fill are calculated, set between 20 and 30% of the landslide area, and the center coordinates of the landslide area are used to overlay non-landslide features onto the landslide region. (5) Result Image Saving. The processed image is saved to a specified path for subsequent model training. The imagery processed by CenterRep is shown in Figure 3. This approach not only retains the overall structure of the landslide images but also enables the model to learn more complex landslide features rather than relying solely on simple visual cues. Simultaneously, by altering the landform attributes of specific areas, this method increases the diversity and complexity of the data, thereby potentially enhancing the model’s generalization ability and edge extraction effectiveness during training.

### 2.3. Architecture of the Proposed Framework

**Figure 4:**
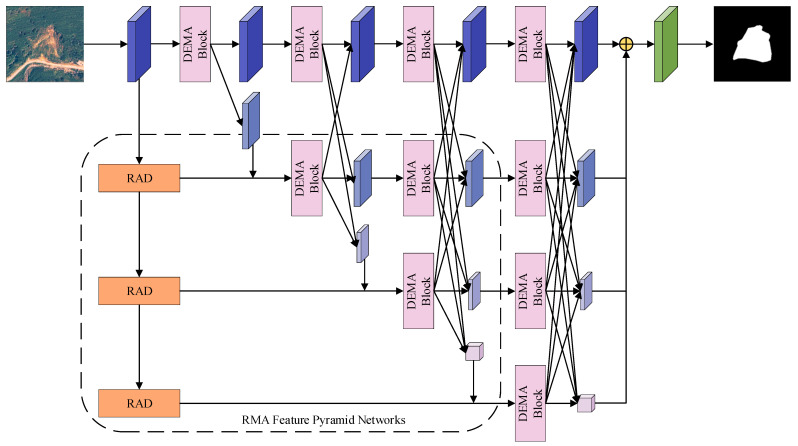
EMR-HRNet model structure.

In traditional semantic segmentation tasks, an encoder–decoder structure coupled with convolution operations is commonly employed for continuous downsampling of images. This process deals with the contextual semantic information in low-resolution images, eventually restoring them to their original high-resolution output [43]. However, with an increasing number of convolutional layers, this approach fails to maintain high-resolution feature information. Moreover, feature extraction through sequential encoders leads to information loss and resource wastage. As information traverses multiple layers, each layer only accesses limited information from its predecessor, leading to a progressive decrease in the amount of information received in subsequent layers, which may result in the loss of critical edge and contour information. In contrast to traditional methods, HRNet adopts an innovative parallel structure that replaces the conventional resolution-reducing approach with a resolution-maintaining operation. In HRNet, high-resolution and low-resolution feature maps continuously exchange information and are processed synchronously. The presence of high-resolution images ensures a more precise spatial resolution, whereas low-resolution images provide more comprehensive semantic information. In this study, we introduce a novel Dilated Efficient Multi-Scale Attention block (DEMA block) to enhance the model’s ability to capture landslide features at different scales, thereby achieving more comprehensive recognition of various types and sizes of landslides. Additionally, HRNet is combined with a feature pyramid structure to effectively preserve spatial feature information at each layer of the model and produce more accurate prediction maps. This approach helps overcome the issue of blurred boundaries, enabling the model to more accurately recognize and delineate the details of landslides. The structure of the proposed model, as shown in Figure 4, demonstrates the advantages of our method in processing spatial details and retaining key information.

### 2.4. RefConv and Multi-Dconv Head Transposed Attention (RMA) Feature Pyramid Structure

**Figure 5:**
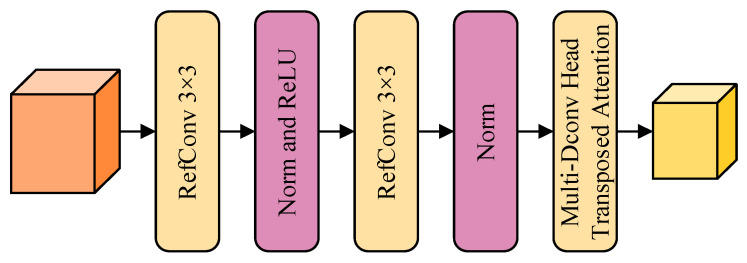
RAD block.

Due to the insufficiency of solely relying on deep feature information to reconstruct accurate prediction maps, it becomes necessary to integrate multi-scale feature maps to compensate for the coarseness of local feature information, thereby achieving precise pixel classification [44]. As a key component in object recognition models, the feature pyramid utilizes multi-level features of an image, providing ample spatial information for each layer of the network to facilitate precise pixel detection and classification [45]. Thus, to enhance the network’s multi-level representational capability, we introduce a pyramid feature layer, which includes the RefConv Attention Downsampling (RAD) block, as depicted in Figure 5.

**Figure 6:**
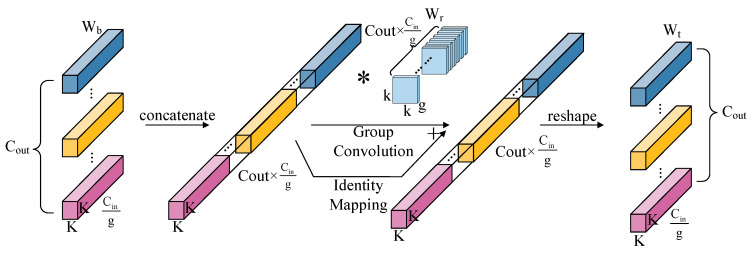
RefConv structure. The * symbol indicates the convolution operator.

The top-down RMA pyramid structure, realized through the RAD block, is employed. RAD comprises two 3 × 3 Re-parameterized Refocusing Convolution (RefConv) layers, batch normalization operations, ReLU activation functions and Multi-Dconv Head Transposed Attention. The RefConv added to the RAD block has the structure shown in Figure 6 [46].

RefConv is an optimized convolution operation designed to augment the convolutional neural network’s focus on key features while reducing interference from irrelevant features. In traditional convolutional neural networks, convolutional layers detect and extract local features from input feature maps by convolving with a set of convolutional kernels. However, this conventional convolutional method may encounter issues when dealing with data that have complex structures and diversity. RefConv addresses these issues by re-parameterizing the input feature maps. It introduces an additional learnable parametric matrix to re-parameterize the input feature map into a new feature map that is better suited to the detection capabilities of the convolution kernels, thereby enhancing the network’s ability to process complex and diverse data. The core idea is to optimize the convolution layer’s response through re-parameterization and focusing mechanisms, enabling the model to learn more effectively. The operation of RefConv can be expressed as shown in Equation (1).

(1) R(X)=Conv(W⊙F,X)

where *R*(*X*) represents the feature map processed by RefConv, *W* denotes the weight of the convolution kernel, *F* is the focusing function used to reweight the convolution kernels and *X* is the input feature map. In RefConv, the base weights $W_{b}$ and transformed weights $W_{t}$ can be represented as $W_{b},W_{t}\in R^{C_{out}\times \frac{C_{in}}{g}\times K\times K}$, with base weights $W_{b}$ transformed into a “feature” $R^{\left(C_{out}\times \frac{C_{in}}{g}\right)\times K\times K}$ through concatenating. These “features” are divided into $G=\frac{C_{out}C_{in}}{g^{2}}$ groups, each with a channel number g and dimension $R^{g\times K\times K}$. The transformed weights $W_{r}\in R^{g\times g\times k\times k}$ modify $W_{b}$, resulting in output dimension $R^{\left(C_{out}\times \frac{C_{in}}{g}\right)\times K\times K}$, and ultimately, the output is reshaped into the transformed weight $W_{t}\in R^{C_{out}\times \frac{C_{in}}{g}\times K\times K}$. Given the complexity of terrain features and various noises typically present in remote sensing imagery, RefConv, with its focusing mechanism, aids the model in more effectively recognizing and extracting features related to landslides while suppressing background noise and irrelevant information.

**Figure 7:**
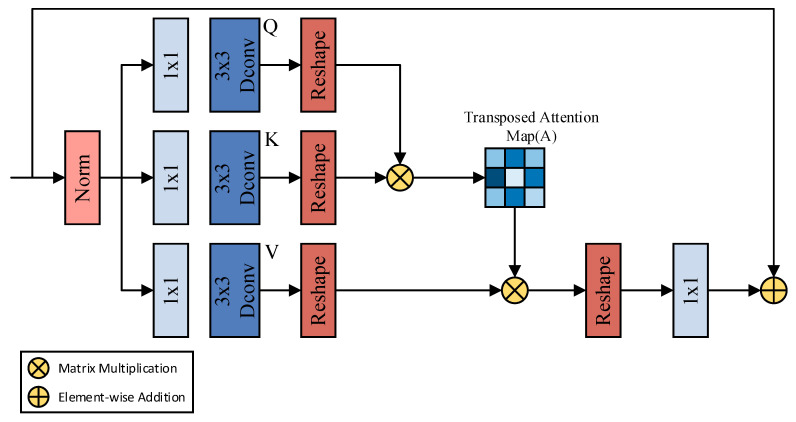
Structure of the MDTA attention mechanism.

Additionally, we incorporate MDTA within the RAD block, as shown in Figure 7 [47]. MDTA is an efficient attention mechanism initially designed for image restoration to address efficiency and performance issues in high-resolution image restoration tasks. In the context of remote sensing imagery for landslide segmentation, we believe MDTA is equally effective. MDTA combines Depth-wise Convolution (Dconv) and Transposed Attention mechanisms to enhance computational efficiency and capture detail.

MDTA aggregates local and non-local pixel interactions, further effectively processing high-resolution images. This method significantly reduces the number of parameters and computational complexity while maintaining the feature extraction capability of convolutional neural networks. The MDTA input feature map $F_{0}\in R^{C\times H\times W}$ first undergoes layer normalization, using 1 × 1 convolution to encode the channel context and 3 × 3 Depth Convolution to aggregate the per-pixel cross-channel context. After the reshape operation, the matrices $Query\in R^{c\times H\times W}$, $Key\in R^{N_{h}\times L\times N_{c}}$ and $Value\in R^{N_{h}\times N_{c}\times L}$ are obtained, where L=H×W; N_h_ and N_c_ represent the number of heads and channels per head, respectively, with channel number $C=N_{h}\times N_{c}$. The output of MDTA, $F_{\mathrm{MDTA}}\in R^{C\times H\times W}$, can be represented by Equation (2).

(2) $$F_{\mathrm{MDTA}}={\mathrm{Conv}}_{1\times 1}\left(Value⊙Softmax\left(Query⊙\frac{Key}{\alpha }\right)\right)+F_{0}$$

where Conv_1×1_ denotes 1 × 1 convolution and α is a learnable scaling parameter. We believe that introducing MDTA into the RMA pyramid structure enables the model to further effectively integrate contextual information across different scales, capturing complex spatial relationships and detail features, thus allowing the model to perform better in recognizing and analyzing complex surface features of landslides. Each scale level’s MDTA, by reinforcing local and global dependencies, enhances the richness of feature expression.

In addition to RefConv and MDTA, the introduced RMA structure allows the model to explore semantic features at different levels, thereby conveying rich multi-level information to the model.

### 2.5. Dilated Efficient Multi-Scale Attention (DEMA) Block

**Figure 8:**
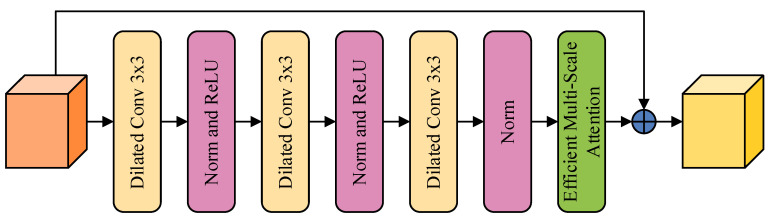
DEMA block structure.

In the parallel structure of the HRNet encoder, the generation of features at different scales enhances high-resolution expressions, thereby improving the overall performance of the model. However, the shallow feature maps in this structure, with their smaller receptive fields, may lead to inconsistencies in class homogeneity within segmentation results. To provide richer feature information at shallow levels, we propose a novel DEMA block designed to integrate feature information from various layers of the original backbone network, significantly expanding the capture range of the receptive field and the model’s ability to discriminate objects of different sizes and shapes, as shown in Figure 8 [48].

Dilated convolution [49] expands the receptive field of the convolution kernel by inserting spaces in the spatial domain, a strategy that neither increases the number of parameters nor the computational burden. For remote sensing imagery, dilated convolution implies capturing broader information while maintaining details. Considering the varied textures and shapes of landslides in remote sensing imagery, we deem dilated convolution an effective method for handling such complex scenarios. Additionally, the DEMA block includes residual connections, further preserving spatial feature information passed from previous network layers. Thus, it enriches the spatial feature information of the receptive field and effectively compensates for local feature loss potentially caused by sparse sampling in dilated convolution, avoiding grid effects.

**Figure 9:**
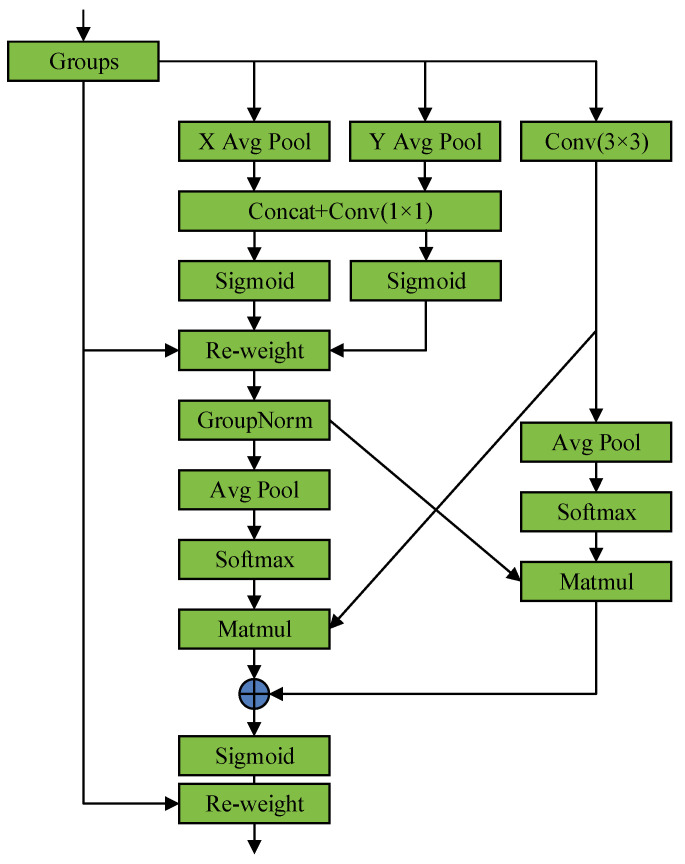
Structure of the EMA attention mechanism.

Furthermore, we integrate Efficient Multi-Scale Attention (EMA) into the DEMA block to enhance the weighting of landslide features and reduce the weighting of irrelevant features, thereby improving the model’s recognition of landslides, as shown in Figure 9. The EMA attention mechanism primarily consists of a 1 × 1 branch, a 3 × 3 branch and a cross-space learning module. EMA first divides the input feature map along the channel dimension into G sub-features, represented as $X=\left[X_{0},X_{i},\ldots ,X_{G-1}\right]$ and $X_{i}\in R^{c//G\times H\times W}$. In the 1 × 1 branch, EMA uses two 1D global average pooling operations along two directions to encode the channels, modeling cross-channel interaction information. The encoded channel features are concatenated along the image height direction, sharing a 1 × 1 convolution kernel, with the output decomposed along the H and W directions into two vectors after a Sigmoid activation function to fit the linear convolution after 2D Binormial, and they are then re-weighted for adaptive feature variable selection as the output of the 1 × 1 branch. The 3 × 3 branch uses a single 3 × 3 convolution to extract multi-scale features as the output of the 3 × 3 branch. The cross-space learning module uses two-dimensional global average pooling to encode the global information of the 1 × 1 branch output, activated by the Softmax function, and the dot product with the output of the 3 × 3 branch, yielding the first spatial attention map. Similarly, the output of the 3 × 3 branch undergoes two-dimensional global average pooling (2D global average pooling) for global information encoding, activated by the Softmax function, and the dot product is used with the group normalized output of the 1 × 1 branch. This yields the second spatial attention map, which retains precise spatial location information with two-dimensional average pooling, as shown in Equation (3).

(3) $$z_{c}=\frac{1}{H\times W}\sum_{j}^{H}\sum_{i}^{W}x_{c}\left(i,j\right)$$

*H* and *W* represent the height and width of the feature map, and *x_c_* denotes the feature tensor of different channels. The first spatial attention map, aggregated and activated by the Sigmoid function and re-weighted for adaptive feature variable selection, yields global context information. The EMA attention mechanism uses 3 × 3 and 1 × 1 convolutions in parallel, utilizing more context information in the intermediate features and fusing different scale context information so that the EMA attention mechanism can generate better pixel-level attention on deep feature maps. The parallelization of convolution kernels can handle short- and long-range dependencies through cross-space learning methods, thus yielding more context relations between features. We believe that, for landslide segmentation tasks, the introduction of EMA can enhance the model’s capture of local details, which is crucial for identifying precise locations and morphologies of landslides. Additionally, as EMA weighs spatial features, the model can focus more on key areas in the imagery, such as potential landslide-active areas.

In summary, by introducing the DEMA block and dilated convolution, this study’s approach not only improves the perception of complex terrain features in remote sensing imagery but also optimizes the model’s detail capture and spatial information integration in landslide segmentation tasks. The fusion of these technologies provides a solid technical foundation for high-precision and efficient landslide segmentation.

## 3. Experiments

In this section, we first introduce the experimental setup and performance evaluation metrics. Subsequently, the landslide segmentation capability is assessed on both the Bijie dataset and the Sichuan and surrounding area dataset, accompanied by ablation experiments to demonstrate the model’s recognition ability.

### 3.1. Experimental Environment and Evaluation Metrics

The experiments were conducted using the PyTorch framework, with Python version 3.6, on an NVIDIA RTX 3080 (10 GB) (Nvidia, Santa Clara, CA, USA) graphics card and 32 GB of memory. During the model training phase, SGD was utilized as the optimizer, with an initial learning rate set at 0.001. A simulated cosine annealing decay strategy was applied to adjust the network learning rate. The batch size during training was uniformly set at 4, with the epochs set to 100. Early stopping was implemented to prevent overfitting. The training data sources included the Bijie dataset and the landslide dataset from Sichuan and its surrounding areas. Specifically, the Bijie dataset contained 770 images, and the Sichuan and surrounding area dataset included 107 images. Following the application of the proposed CenterRep data augmentation method and other common data augmentation techniques, such as rotation and brightness adjustments, the Bijie dataset was expanded to 5720 images, and the Sichuan and surrounding dataset was expanded to 804 images, with all images resized to 512 × 512 pixels. To prevent potential biases or overfitting due to data augmentation, dropout and L2 regularization were incorporated into the model. Dropout layers were added after several layers of the model, with a dropout rate of 0.5. During training, a weight decay parameter was set in the optimizer to implement L2 regularization. Moreover, the diversity of the data augmentation methods prevented the model from learning spurious correlations, ensuring robust training. Both datasets were split into training, validation and testing sets with a 6:2:2 ratio, with the training set used for network weight training, the validation set used to assess the effectiveness of the training process and determine the need for weight updates and the testing set used to evaluate the model’s performance in landslide detection tasks. Using these two datasets, training and comparison were conducted on EMR-HRNet, the original HRNet model and five other models (DeeplabV3, PSPNet, Segformer, UNet and SE-Unet). Additionally, to validate the effectiveness of our proposed modules, ablation experiments were conducted on the RMA block, DEMA block and data augmentation method.

To systematically compare the model’s recognition performance, six widely used metrics were adopted to measure the performance of all semantic segmentation models, namely mIoU, precision, recall, accuracy and F1 score. The definitions of these metrics are shown in Equations (4)–(9).

(4) $$\mathrm{IoU}=\frac{\mathrm{TP}}{\left(\mathrm{FN}+\mathrm{FP}+\mathrm{TP}\right)}$$

(5) $$\mathrm{mIoU}=\frac{1}{2}\left({\mathrm{IoU}}_{\mathrm{landslides}}+{\mathrm{IoU}}_{\mathrm{background}}\right)$$

(6) $$\mathrm{Precision}=\frac{\mathrm{TP}}{\left(\mathrm{TP}+\mathrm{FP}\right)}$$

(7) $$\mathrm{Recall}=\frac{\mathrm{TP}}{\left(\mathrm{TP}+\mathrm{FN}\right)}$$

(8) $$\mathrm{Accuracy}=\frac{\mathrm{TP}+\mathrm{TN}}{\mathrm{TP}+\mathrm{TN}+\mathrm{FP}+\mathrm{FN}}$$

(9) $$F1\_\mathrm{score}=\frac{2\times \mathrm{Precison}\times \mathrm{Recall}}{\mathrm{Precision}+\mathrm{Recall}}$$

TP (true positive), FP (false positive), TN (true negative) and FN (false negative) rates can be calculated using a confusion matrix. Overall performance is assessed through the mIoU, precision, recall, accuracy and F1 score for both landslide and background classes. For all metrics in the equations, higher values indicate superior recognition performance.

### 3.2. Results for the Bijie Dataset

Initially, the performance of EMR-HRNet was tested using the Bijie dataset and compared against other advanced models (DeepLabV3, PSPNet, SegFormer, Unet (VGG), HRNet and SE-Unet). As indicated in Table 2, the EMR-HRNet model achieved the highest mIoU score, followed by SE-Unet, HRNet, PSPNet, DeepLabV3, Unet (VGG) and SegFormer. Notably, SegFormer, a transformer-based semantic segmentation model, did not perform as expected in the landslide segmentation task, scoring 5.95% lower in mIoU than that of EMR-HRNet. Furthermore, the improved Unet (SE-Unet) model displayed a remarkable mIoU score of 81.52% in the Bijie dataset, which was only slightly lower than that of EMR-HRNet. In terms of precision and recall scores, although EMR-HRNet did not achieve the highest values, it still ranked among the top out of all the models. The EMR-HRNet model scored the highest in accuracy, reaching 94.68%, surpassing the other models. Furthermore, the average F1 scores for DeepLabV3, PSPNet, SegFormer, Unet (VGG), HRNet and SE-Unet were 88.57%, 88.43%, 85.22%, 86.19%, 88.43% and 89.44%, respectively, which were all lower than those of EMR-HRNet. Compared to the original HRNet and SE-UNet, EMR-HRNet’s performance was higher by 1.07% and 0.06%, respectively. Despite achieving the highest values in mIoU and F1 score, the score gap was not significant, which, upon analysis, we attribute to the characteristics of the Bijie dataset. As satellite imagery, its details might not be sufficient to exhibit significant recognition differences in the model.

**Figure 10:**
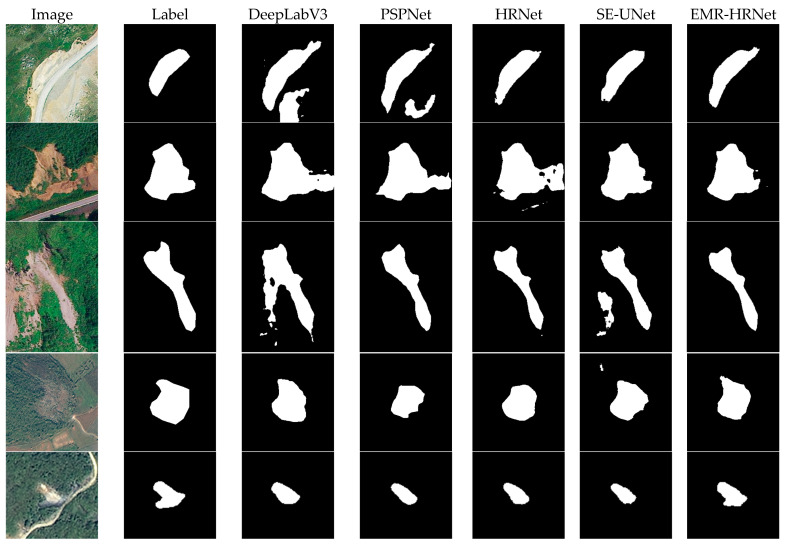
Bijie dataset visualization results.

As shown in Figure 10, the models DeepLabV3, PSPNet, HRNet, SE-Unet and EMR-HRNet exhibit varying degrees of effectiveness in identifying landslides of different shapes. It was observed that the PSPNet and HRNet models experienced void extraction and a certain degree of “salt-and-pepper effect,” leading to varying extents of missed extractions. The DeepLabV3, PSPNet, HRNet and SE-UNet models were prone to misidentification in regions with colors similar to landslides. Furthermore, compared to the EMR-HRNet model, the edge extraction of other models lacked clarity, failing to distinctly recognize the features of landslide edges. In contrast, our model’s segmentation results included more detailed features, with clearer and more precise edge recognition. This indicates that the DEMA block introduced in this article enhanced the model’s detail capturing ability, significantly improving edge recognition in landslide identification. This finding is crucial, as it highlights the importance of enhancing a model’s capability to process intricate terrain features in semantic segmentation. The introduction of the DEMA block provides the model with a deeper and more nuanced understanding of the environment, which is essential for achieving high-precision landslide detection.

### 3.3. Results for the Sichuan and Surrounding Area Dataset

With the widespread availability of consumer-grade Unmanned Aerial Vehicles (UAVs), UAV remote sensing imagery has become increasingly prevalent in recent years. Offering resolutions up to the centimeter level, it provides the capability to capture detailed information about landslides, making it highly valuable for precise landslide segmentation. Therefore, this paper conducted the same tests on UAV landslide imagery across different models as those conducted on the Bijie dataset, and the results are presented in Table 3. PSPNet, utilizing pyramid pooling modules to capture contextual information at various scales, achieved an mIoU score of 59.02% in the dataset. The original HRNet model, which changes the connection between high and low resolutions from series to parallel and maintains high-resolution features throughout the network structure, performed better on the information-rich UAV imagery, reaching an mIoU score of 59.38%. SE-UNet, enhanced with SENet attention modules in the model, also achieved notable recognition results, with an mIoU of 63.17%. Our proposed model achieved the best metrics with the Sichuan dataset, with the EMR-HRNet model’s mIoU, precision, recall, accuracy and F1 scores being 71.68%, 81.12%, 85.03%, 87.71% and 83.03%, respectively, ranking the best in all model assessments. Compared to the highest-scoring SE-UNet model from the other models, our mIoU was still 8.51% higher. This significantly demonstrates our model’s superior learning and recognition capabilities when dealing with imagery containing more information and a higher resolution.

**Figure 11:**
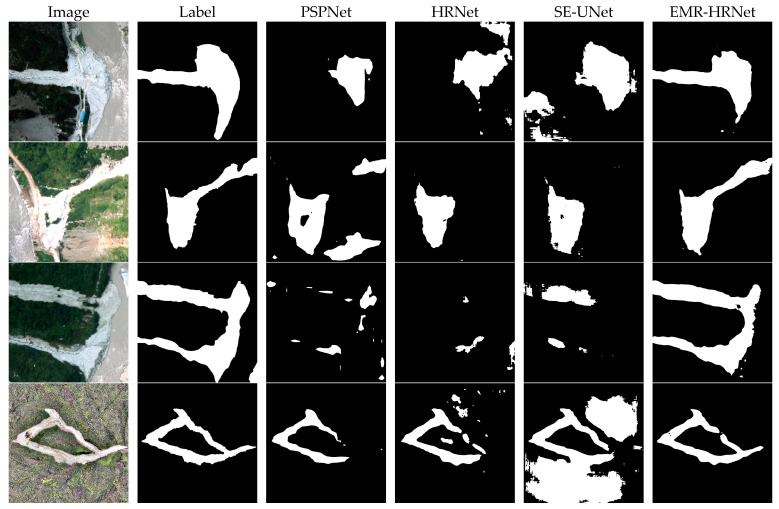
Sichuan and surrounding area dataset visualization results.

Due to the subpar test results of DeepLabV3, SegFormer and UNet (VGG) in this dataset, they were not displayed in the visual representation. As seen in Figure 11, our proposed model showed better performance in landslide detection with high-resolution UAV imagery. Although PSPNet, the original HRNet and SE-UNet achieved higher scores in various metrics among the advanced models, they all exhibited varying degrees of false positives and negatives when dealing with complex background imagery, with suboptimal extraction effects and even failing to accurately extract basic landslide outlines. In contrast, EMR-HRNet, although occasionally displaying the “salt-and-pepper effect”, was able to extract more accurate landslide targets for most landslides. This efficacy is attributed to the learning of complex features in data augmentation and the introduction of the RMA pyramid structure, which aids the model in more effectively recognizing features related to landslides and suppressing background noise. Additionally, the MDTA within the RMA pyramid structure further enables the model to effectively fuse contextual information at different scales and capture complex spatial relationships and details. Hence, our model demonstrated superior capabilities in identifying the contours and edges of landslides. Integrating these advanced modules into the HRNet model significantly enhanced its segmentation capabilities, especially in accurately extracting landslide edges against complex backgrounds. These improvements offer an effective technical approach for landslide recognition tasks with high-resolution UAV imagery, underscoring the importance of precise surface feature segmentation in complex environmental settings.

### 3.4. Ablation Experiments

To verify the effectiveness of the CenterRep data augmentation method, the RMA pyramid structure and the DEMA block in EMR-HRNet, ablation experiments were conducted on the combined dataset of Bijie and Sichuan and the surrounding area, totaling 6524 images. The dataset was split into training and testing sets with a 4:1 ratio, and mIoU was used as the assessment metric. The results of these experiments are shown in Table 4.

In the ablation experiments, the original HRNet model served as the baseline for comparison, and HRNet was optimized using various combinations of CenterRep data augmentation, the RMA pyramid structure and the DEMA block, resulting in five additional models for comparison. First, we validated the performance of the DEMA block, which replaced the 3 × 3 convolution layers in the backbone network of the HRNet model, expanding the receptive field without sacrificing spatial resolution and enhancing the model’s ability to capture local details. Additionally, the RMA pyramid structure was incorporated into the experiments, adding RefConv and MDTA to facilitate more effective recognition and extraction of landslide-related features. The results, as seen in Table 4, indicate that introducing the DEMA block and RMA structure, along with training using CenterRep-processed data, improved the mIoU from 66.36% to 72.53%. This significant increase demonstrates that data processed by CenterRep enables the model to learn deeper landslide features, thereby enhancing the model’s robustness and generalization capability. Overall, our experimental results strongly suggest that the EMR-HRNet model excels in recognizing landslide features in various types of imagery. The integration of a series of innovative structures and methods not only optimizes the accuracy of surface feature recognition but also improves the model’s generalization ability under complex terrain conditions, providing robust technical support for landslide recognition research.

## 4. Discussion

### 4.1. Universal Experiment

Experimental outcomes from prior studies on two public datasets have substantiated the effectiveness of incorporating an RMA pyramid structure within the model. This integration proficiently amalgamates contextual feature information while concurrently mitigating background noise, thus enhancing the model’s aptitude for target information acquisition. Further, the addition of the EAD block augments the model’s intra-class consistency and bolsters its capacity for detailed local feature detection. Image data processed using CenterRep data augmentation not only diversified the dataset but also facilitated more comprehensive learning of landslide details. EMR-HRNet’s performance, as evidenced by achieving the highest mIOU and F1 scores in both the Bijie and the Sichuan and surrounding area datasets, underscores its exceptional capability for landslide detection in remote sensing imagery. Subsequent validation employed the Luding County dataset, encompassing 230 images sourced from Google Earth data spanning 2020–2022 with ground resolutions of 0.4–0.6 m. The landslides contained rockfalls, debris flows and other types of landslides, which further attests to EMR-HRNet’s robustness in this domain [50].

**Figure 12:**
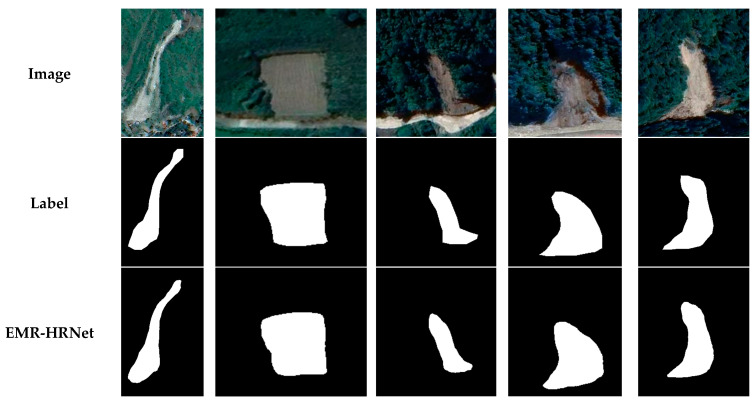
Luding County dataset visualization results.

Figure 12 illustrates EMR-HRNet’s proficiency in accurately demarcating primary landslide regions, significantly reducing false extractions, omissions and the “salt-and-pepper effect”. This demonstrates the model’s adeptness in landslide detection across both satellite and drone imagery. However, the model exhibits limitations in precise edge extraction for shadow-obscured landslide areas within the Luding County dataset, a challenge likely influenced by data spatial resolution constraints. Despite these limitations, the overall detection results clearly delineate landslide area contours, confirming the method’s practical relevance in landslide recognition within Google Earth imagery. Although the model has shown advancements in boundary recognition, the irreversible nature of information loss during downsampling remains a challenge. Future research will focus on enhancing the precision of landslide edge detection in shadow-affected remote sensing images.

Additionally, experiments utilizing the EMR-HRNet model for training and landslide recognition testing on the Bijie dataset, the Sichuan and surrounding area dataset and the Luding County dataset have shown good test results. These datasets include various types of landslides, such as rockfalls, debris flows and rainfall landslides. The experimental results demonstrate that the EMR-HRNet model exhibits excellent recognition performance under diverse environments and conditions, proving the model’s applicability and robustness under extensive geographical conditions.

### 4.2. Comparison with Different Blocks

This section compares the performance of the HRNet models based on different blocks. Four models were trained using the same dataset: HRNet + DEMA, HRNet + GAM, HRNet + ParNetA and HRNet + CBAM. The training results are displayed in Table 5. The table shows that, compared to other blocks, the DEMA block exhibits superior performance. This advantage primarily stems from the DEMA block’s ability to effectively expand the receptive field and capture local features accurately when processing remote sensing imagery with complex textures and morphologies. The DEMA block utilizes dilated convolution to not only enhance the model’s spatial perception but also, through an effective multi-scale attention mechanism, further improves the model’s ability to recognize landslide features. Therefore, the DEMA block was selected as the model block for this study.

### 4.3. Comparison with Previous Work

Recently, some researchers have successfully applied deep learning methods to landslide identification and extraction, achieving favorable results. Yang [51], using UNet, DeepLabV3+ and PSPNet, conducted identification and semantic segmentation of landslides in the Bijie area, demonstrating the feasibility of deep learning in landslide detection. However, when facing more complex landslide scenarios, basic deep learning models still exhibit some shortcomings, prompting scholars to improve various deep learning models to enhance landslide segmentation. Lv [42] proposed a landslide identification model named ShapeFormer, incorporating a pyramid vision transformer (PVT), which yielded better results than those of the basic models. Zhang [52] developed a dynamic module, FFEM, based on structural reparameterization theory, which reconstructed the decoder of the UNet model. Moreover, several other researchers have made improvements based on different models to better perform landslide recognition tasks [53]. A comparison of this paper’s model with previous work is shown in Table 6.

Table 6 indicates that, compared to previous work, the EMR-HRNet model presented in this paper achieves the highest scores for all evaluation metrics. This is because the approach introduced by this paper uses CenterRep data augmentation to enable the model to learn more complex features in real-world landslide detection tasks. Additionally, the RMA and DEMA structures expand the model’s receptive field and enhance its capability for semantic recognition and expression at various levels. In contrast, existing research lacks attention to important landslide features, resulting in suboptimal recognition performance. Therefore, the methods proposed in this study can identify landslides more accurately.

### 4.4. Limitations and Future Work

Despite the exemplary recognition accuracy of EMR-HRNet in both datasets, there is room for further optimization. To assess model performance more deeply, we compared the number of trainable parameters and Floating Point Operations Per Second (FLOPs) processed by each model, with details presented in Table 7.

Among these models, PSPNet, DeepLabV3 and SegFormer demonstrated higher efficiency in several models, yet their accuracy remained lower than that of our proposed model. Notably, compared to the original HRNet, the increase in trainable parameters for EMR-HRNet was minimal, indicating a focus on model compactness while maintaining accuracy. Moreover, compared to UNet (VGG), EMR-HRNet exhibited a significantly higher mIoU in the Sichuan and surrounding area dataset without introducing additional computational complexity. These results highlight the significant advantages of EMR-HRNet over existing advanced models. However, despite its superior recognition capabilities, our model is not as lightweight compared to others. In future work, we plan to incorporate various lightweight modules into the model to make it more efficient while maintaining performance. Additionally, although our model has shown good performance in UAV landslide imagery, we aim to acquire more UAV landslide images in the future, which will help improve the model’s recognition generalization ability for landslide imagery captured by different sensors. In this way, the model will not only be able to handle images in the current dataset but will also be able to adapt to diverse environments and conditions, enhancing its usability and robustness in practical applications.

## 5. Conclusions

In this paper, we primarily focus on the problem of landslide detection in optical remote sensing imagery using deep learning methods. We analyze the structure and limitations of the HRNet model and the characteristics of remote sensing imagery and propose solutions from different perspectives. We introduce a data augmentation strategy, CenterRep, specifically for landslide imagery, which not only enhances data diversity but also enables the model to learn more complex landslide features. For the HRNet model, we incorporate the EMR pyramid structure and the DEMA block. The introduction of the EMR pyramid structure effectively combines contextual information at various scales, capturing a range of features while suppressing background noise and irrelevant information. Moreover, the inclusion of the DEMA block enriches the model’s receptive field and spatial feature information, thereby enhancing its ability to capture local details while avoiding grid effects. We evaluate the proposed strategies and models on the Bijie and Sichuan and surrounding area datasets, conducting extensive comparative experiments. The results demonstrate that our EMR-HRNet model achieves the highest mIoU values and F1 scores in landslide segmentation tasks in both satellite and UAV imagery, compared to other models. This validation of the method’s effectiveness and universality confirms that our proposed model is reliable and meets the needs for accurate identification of landslide disaster information.

## Figures and Tables

**Table 1:** Dataset image parameters.

Dataset Name	Bijie Dataset	Sichuan and Surrounding Area
Data Type	Satellite Image	UAV Image
Data Source	TripleSat	UAV
Spatial Resolution (m)	0.8	0.2–0.9
Acquisition Time	May–August 2015	2008–2020
Location	Bijie City, Guizhou Province	Wenchuan County, Pingwu County, Maoxian County, Sichuan Province
Landslide Types	Debris Avalanche, Rock Slides, Rockfalls	Earthquake Landslides, Rainfall Landslides, Gully Debris Flows, Slope Debris Flows
Number of Bands	3 (Red, Green, Blue)	3 (Red, Green, Blue)

**Table 2:** Results of the Bijie dataset.

Method	mIoU (%)	Precision (%)	Recall (%)	Accuracy (%)	F1 (%)
DeeplabV3	79.80	85.20	92.21	93.27	88.57
PSPNet	80.20	87.57	89.30	94.28	88.43
SegFormer	75.75	86.28	84.19	93.06	85.22
Unet (VGG)	77.03	87.90	84.55	93.59	86.19
HRNet	80.24	87.98	88.88	94.35	88.43
SE-Unet	81.52	87.38	91.60	94.55	89.44
EMR-HRNet	81.70	87.91	91.15	94.68	89.50

**Table 3:** Results of the Sichuan and surrounding area dataset.

Method	mIoU (%)	Precision (%)	Recall (%)	Accuracy (%)	F1 (%)
DeeplabV3	57.35	70.31	77.57	77.13	73.76
PSPNet	59.02	71.61	73.49	80.72	72.54
SegFormer	58.84	71.18	74.37	80.08	72.74
Unet (VGG)	58.40	73.41	69.90	82.18	71.61
HRNet	59.38	72.39	72.83	81.48	72.61
SE-UNet	63.17	74.83	77.86	82.99	76.31
EMR-HRNet	71.68	81.12	85.03	87.71	83.03

**Table 4:** Results of ablation experiments.

Baseline	DEMA	RMA	CenterRep	mIoU (%)
√				60.99
√	√			63.51
√		√		62.35
√	√	√		66.36
√	√		√	70.17
√	√	√	√	72.53

**Table 5:** Comparison of different blocks.

Model	mIoU (%)	Precision (%)	Recall (%)	Accuracy (%)	F1 (%)
HRNet + GAM	60.71	72.92	74.96	82.16	73.93
HRNet + ParNetA	61.46	74.29	74.32	83.24	74.30
HRNet + CBAM	62.52	74.77	75.92	83.53	75.34
HRNet + DEMA	63.51	75.05	77.89	83.63	76.44

**Table 6:** Comparison with other improved models.

Model	mIoU (%)	Precision (%)	Recall (%)	Accuracy (%)	F1 (%)
DeepLabV3+	75.99	84.43	88.37	\	86.35
TransUNet	78.15	87.24	88.23	\	87.73
ShapeFormer	78.72	86.74	89.52	\	88.11
Re-Net	72.23	\	\	\	83.88
EMR-HRNet	81.70	87.91	91.15	94.68	89.50

**Table 7:** Comparison of the efficiency of different models.

Method	mIoU (%)	FLOPs (G)	Parameters (M)
DeeplabV3	57.35	53.03	5.82
PSPNet	59.02	6.03	2.38
SegFormer	58.84	13.67	3.72
Unet (VGG)	58.4	452.31	24.89
HRNet	59.38	80.18	29.55
EMR-HRNet	71.68	82.15	31.21

## Data Availability

All datasets used in this study are publicly available. The Bijie dataset is available from http://gpcv.whu.edu.cn/data/Bijie_pages.html (accessed on 10 December 2023). Data for Sichuan and surrounding areas are available from https://www.scidb.cn/en/detail?dataSetId=803952485596135424 (accessed on 10 December 2023). Luding County landslide dataset download link: https://pan.cdut.edu.cn/#/link/B007C24A04BAC995CC7D782DE0483C8F?_k=jxa1pj (accessed on 25 March 2024).
